# Genome resource announcement of a *Leptodophora* sp. fungus isolated from roots of broadleaf plants in Wisconsin, USA

**DOI:** 10.1128/mra.00936-24

**Published:** 2025-07-23

**Authors:** Tristan Yang, Amanda Certano, Venkatachalam Lakshmanan, Sebastian Bassi, Neeraj Purushotham, Tegan Nock, Abed Chaudhury, Animesh Ray

**Affiliations:** 1 Loam Bio, Orange, New South Wales, Australia; 2 Riggs School of Applied Life Sciences, Keck Graduate Institute 48927, Claremont, California, USA; 3 Division of Biology and Biological Engineering, California Institute of Technology 6469, Pasadena, California, USA; University of Maryland School of Medicine, Baltimore, Maryland, USA

**Keywords:** fungi, genome, whole genome, soil fungi, endophyte, long read, short read

## Abstract

We report whole genome sequences of a plant root-associated fungus *Leptodophora* sp., consisting of 21 contigs representing 75.14 mega-base pairs genome encoding 19,447 proteins, with 98.4% BUSCO proteome completeness score. Secondary metabolism genes constitute 55 clusters. This species likely synthesizes toxic alternapyrone, 6-methylsalicylic acid, and fusarin.

## ANNOUNCEMENT

The genus *Leptodophora* (1) (previously, *Cadophora* (2)) contains endophytic fungi known for their non-pathogenic root association. Some members are dark septate endophytes (3) important for plant nutrition, stress, pathogen deterrence, and community dynamics (4).

A pure fungal sample (TYLB-001) was isolated from broadleaf plant roots in Spooner, WI, by surface sterilizing and plating on potato dextrose agar (PDA, Sigma) with Penicillin−Streptomycin−Neomycin (Sigma, P4083) and subculturing (25°C for several weeks). DNA was isolated from liquid cultures by grinding in liquid nitrogen, lysed with SDS (1% v/w), treated with ribonuclease A and proteinase K, and extracted with chloroform:isoamyl alcohol (24:1, v/v); DNA was precipitated in ethanol (5). DNA libraries were prepared at the University of Minnesota Genomics Center (St. Paul, MN; PacBio HiFi long-read sequencing) (6), and at the Australian Genome Research Facility (for Illumina short-read using Nextera DNA Flex Library Preparation kit). Long-read size selection was by High Pass Plus (>15 kb) and sequencing was on PacBio Sequel II with 8M SMRT Cell. HiFi reads were generated using Circular Consensus Sequencing, which are already quality-filtered and error-corrected; additional trimming or correction was unnecessary. A total of 324,971 HiFi reads were generated, with N50 = 15,785 bp.

Illumina paired-end reads were trimmed using fastp v.0.23.2 (7); 20,796,372 paired-end reads were assembled. Jellyfish 2.2.8 (8) and GenomeScope 2.0 (9) estimated initial genome sizes (k-mer frequency analysis). Short-read sequence data captured 96.9% of the genome by GenomeScope v.2.0 model fitting. HiFiasm v.0.19.9-r616 (10) assembled 324,971 long-reads; scaffolded by SSPACE-Long v.1.1 (11); gaps closed by LR_Gapcloser v.2018.9.4 (12); trimmed paired-end reads were aligned to the gap-closed assembly using Burrows-Wheeler transformation algorithm v.0.7.17-r1188, and the resulting SAM file was processed in SAMTools v.1.12. Pilon v.1.24 (13) was used to polish and gap-fill short reads. Repeats were “softmasked” by RepeatMasker v.4.1.2-pl on the DfAM-3.9 (14, 15) “slow search” and “Fungi” species options. The HiFi reads covered 90.8% of the genome.

The final genome was 75,142,167 bp in 21 contigs (N50 = 5,880,590 bp, 45.53% GC). Protein-coding genes were predicted by Funannotate v.1.8.17 (16) with AUGUSTUS (17), GeneMark-ES-2 v.1.14_1.25 (18), SNAP v.2.2.4-14ubuntu2 (19), and GlimmerHMM v.3.0.4 (20). Gene models produced a consensus by Evidence Modeler v.2.1.0 (21). Diamond v.2.1.11 (22) and BEDtools 2.31.0 (23) filtered low-quality models. A total of 19,447 protein-coding genes were found. tRNAscan-SE v.2.0 predicted tRNA genes (24). Phylogenetic analysis was performed using the Internal Transcribed Spacer (ITS) region spanning ITS1F (5′CTTGGTCATTTAGAGGAAGTAA3′) and ITS4 (5′TCCTCCGCTTATTGATATGC3′) aligned via NCBI BLAST. This placed the fungus in the genus *Leptodophora*. BUSCO v.2.0 (25) on dikarya_odb9 (26) produced 98.4% proteome completeness score. We predicted biosynthetic gene clusters with antiSMASH v.6.1.1 (27) and found 55 clusters, including those likely to synthesize alternapyrone, 6-methylsalicylic acid, and fusarin. The complete analytical pipeline is available at https://doi.org/10.17504/protocols.io.e6nvw14nwlmk/v5.

## Data Availability

The GenBank accession number of the project is JBGRZW000000000 (first version, JBGRZW010000000). The raw sequence data were deposited under accessions SRR30545879, SRR30545880, and SRR30545881, associated with BioProject PRJNA1153414 and BioSample SAMN43359310.
